# Some Clinical Cases

**Published:** 1881-07

**Authors:** Edward Rushmore

**Affiliations:** Plainfield, N. J.


### CLINICAL CASES.

EDWARD RUSHMORE, M. D., PLAINFIELD, N. J.

### CASE I. PSORIC IRRITATION OF SPINE AND EYES. CALC., LYC., AGARICUS.

OCTOBER 7TH, 1880. Miss Mary C., aged sixteen, consulted me, with the following history and symptoms: Has had weak eyes from infancy, which have been worse for one month. She menstruated at ten years, when she began to be sick, suffering from spasms of pain, leucorrhœa and numbness of limbs, so that she could not feel the floor on walking. The menses have been irregular—at intervals varying from two to ten weeks, copious and free from pain; the interval is now about three weeks.

She sees only the left half of objects; in place of the right half, bright zigzags; sees bright spots—green, red and white; also, a mist before the eyes, so that distant objects look dim. She suffers much from heat in the eyes and pressure in the back of the orbits; worse from looking fixedly, from reading, from bright light, natural or artificial, from mental exertion, and in the evening. This heat is also worse from closing the lids and in wind, better from cold applications. There is itching of the lids and canthi. She has constant pain in the temples and over the eyes, worse in warm room and weather, from exertion of the eyes and in wind. There is heat, smarting and a sense of sticks and distress in the spine. The backache is worse from standing and walking, and is relieved by pressure in the lumbar region; but pressure in the dorsal region excites laughter.

The local and constitutional symptoms were together regarded as most similar to those of *Calcarea Carbonica*, of which she received Dunham's 200th potency, night and morning for three days.

Improvement set in at once; but delight at being able to use her eyes, led to over-use of them, and the report by letter a month later was, that nausea from use of the eyes, heat of the face, pain in the back from lifting, as if she had no feeling except in the back, coldness between the shoulders, extending to the sides, and cold feet had been added to the former symptoms. She received *Lycopodium* c. m. (Swan), morning and evening for four days. In three weeks, December 1st, her mother wrote that the back was almost well, but that the head and eyes were worse, paining severely and incessantly, with heat in the head and face, so that she could not stay in a room of ordinary warmth. *Phosphorus*, in high potency, was sent her, without benefit, and again *Calcarea*, with but little improvement. She reported again as follows: The eyes worse, the pain constant, deep-seated and sharp shooting, worse in light and from reading, better in cold air and in twilight. Heat in the eyes; they burn on closing the lids and from exertion. The feet are very cold and swollen, with troublesome chilblains. The spine is sensitive to touch in various parts.

The chilblains suggested *Agaricus*, which, on examination, was found to present the principal symptoms of the case. Heat in the eyes, worse from closing the lids, inability to use the eyes, with painful sensitiveness to light; mist before the eyes, dryness, itching of the canthi, soreness of the spine to touch and troublesome chilblains, with cold feet, were all found under *Agaricus*. Accordingly, seven powders of Finch's 900th potency were sent her, with the request to take one every morning. In a few weeks she reported by letter that soon after beginning the last remedy, she got better; that she could use her eyes at any kind of work, and that she seemed in every respect well. Recent inquiry elicited the information that she has remained well.

### Case II. Amenorrhœa. Kali Carbonicum.

In October, 1880, Miss M., aged about eighteen, had missed the menstrual flow for two consecutive periods. She has dark circles around the eyes, bloated, hard abdomen, hawking of mucus and a hacking cough, worse toward evening. She received *Kali Carb.*, c. m. (Swan) one powder. After that, the menses were regular and she well.

### Case III. Indigestion. Carbo Veg.

March 4th, 1880, Mrs. C. complained of nausea and pain in the pit of stomach, worse after eating; the pain extending to left side and abdomen; belching of wind after the pain which relieves; cannot bear pressure of clothes on stomach; complains of greasy tasting water in mouth, worse after eating meat; cannot sleep sound at night and feels unrefreshed in the morning. *Carbo veg.* relieved.

### Case IV. Cough. Phosphorus.

March 31st, 1881, Mr. E. His throat troubles much; has had for some months a hacking cough for an hour in the morning, till he gets his throat cleared of yellowish mucus, slightly blood-streaked, and again at 3 P. M. and at 7 P. M., none at night; the cough is worse from talking and from excitement; it seems to proceed from an irritation deep in the larynx, which feels as if he had swallowed a bristle; there is a slight pain in the throat when coughing, and some gagging and nausea. He wakes at 2 A. M. and remains awake for three hours, then sleeps late in the morning. Has chills running up the back; has taken "sickening medicine" without relief. He received *Nux. Vom.* c. m. (Swan) one dose. In five days he had coughed less, only on the day before. He then received *Phosphorus* c. m. (Swan) one dose. In four days the cough was much better; he slept till six in the morning and had less chilliness in the back. He received no more medicine, and in a few days became and remained free from the cough and other symptoms.

### Case V. Cough. Phosphorus.

March 24th, 1881. Mr. C. has had cough for several weeks, caused by tickling in the chest, worse at midnight, when it nearly chokes him; it hurts his head as if it would split. The expectoration is copious, bluish, salty; the breath is salty; he has vertigo and obstruction of the nose. He formerly had cough only in the summer, but now has it every winter. He received one dose of *Phosphorus* c. m. (Swan) and began to feel better next day, and was well in a few days without more medicine.

